# The CCR4-NOT deadenylase complex mediates tubulin autoregulation *via* specific adapters CNOT10 and CNOT11

**DOI:** 10.17912/micropub.biology.001880

**Published:** 2025-11-18

**Authors:** Stephanie L. Sarbanes, J. Robert Hogg, Antonina Roll-Mecak

**Affiliations:** 1 National Institute of Neurological Disorders and Stroke, Bethesda, Maryland, United States; 2 Biochemistry & Biophysics Center, National Heart Lung and Blood Institute, Bethesda, Maryland, United States

## Abstract

Tubulin autoregulation maintains cellular microtubule homeostasis by triggering rapid degradation of tubulin mRNAs in response to an increase in soluble α- and β-tubulin levels. Through siRNA knock-down of several RNA decay pathways coupled with Roadblock-qPCR kinetic measurements, we independently validate and extend prior work by identifying the CCR4-NOT deadenylase complex components CNOT1, CNOT10, and CNOT11 as central effectors both in tubulin autoregulation and basal tubulin mRNA stability. In contrast, depletion of ribosome quality control and other decay factors has little effect. These findings corroborate CCR4-NOT adaptors as essential effectors of tubulin autoregulation and provide molecular entry points to dissect microtubule homeostasis.

**Figure 1. Tubulin autoregulation is mediated by CCR4-NOT complex and its subunits CNOT10 and 11:**
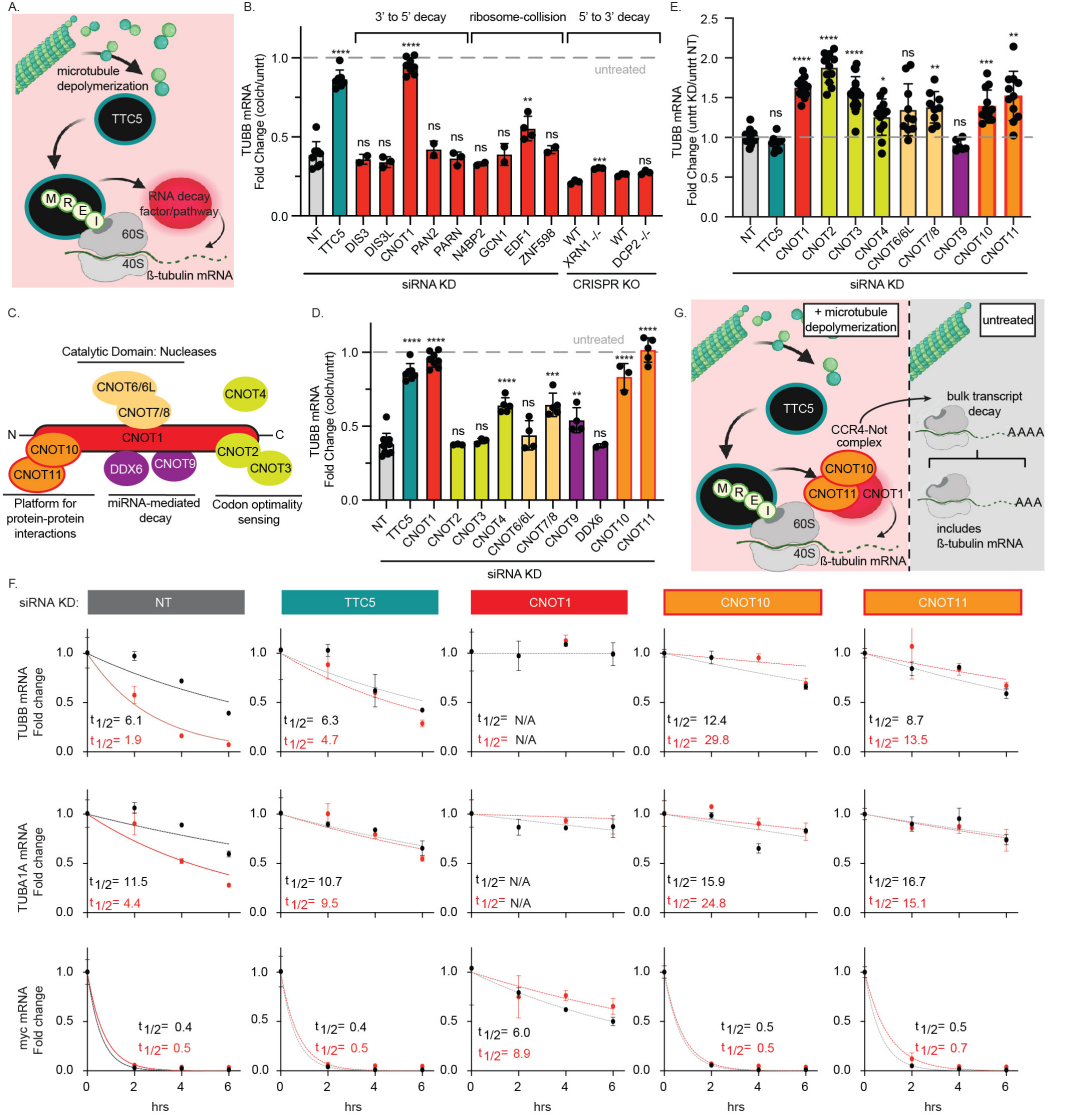
(A) Graphical depiction of tubulin autoregulation—an increase in soluble tubulin due to microtubule depolymerization (such as upon colchicine treatment) results in co-translational degradation of tubulin transcripts *via* TTC5. (B) RT–qPCR analysis of TUBB mRNA expression upon colchicine treatment (10 μM; 4hrs) of either CRISPR gene-edited lines knocked-out for the indicated gene (XRN1-/- in A549, DCP2 -/- in HEK-293) or HEK-293 cells first transfected for 72 hrs with the panel of siRNAs to induce knock-down of the indicated gene. Data are graphed as the fold change in response to colchicine relative to the untreated condition for each knock-out or siRNA KD (preceded by normalization to housekeeping gene Glyceraldehyde 3-Phosphate Dehydrogenase [GAPDH]). For the CRISPR KO lines, each point on the graph reflects three biological replicates. For the siRNA KDs, each point represents an independent experiment comprised of two biological replicates. Error bars indicate mean+/-SD and significance calculated by Student’s two-tailed t-test to non-targeting (NT) control. (C) Graphical depiction of the CCR4-NOT deadenylase complex with associated factors and functions. (D) RT–qPCR analysis of TUBB mRNA expression upon colchicine treatment (10 μM, 4 hrs) following transfection of HEK-293 cells for 72 hrs with the panel of indicated CCR4-NOT-centric siRNAs. Fold change and significance calculated as in (B). (E) Normalization of RT-qPCR data to the NT untreated condition to assess baseline changes in TUBB mRNA levels upon siRNA knock-down of the indicated genes—each point represents an independent replicate across a minimum of 3 independent experiments. Error bars indicate mean +/-SD and significance calculated by Dunnett’s One-way ANOVA to the NT control. (F) Measurement of mRNA decay rates in untreated and colchicine-treated cells by Roadblock-qPCR upon knock-down of tubulin autoregulation-implicated factors. HEK-293 cells were transfected with indicated siRNAs for 72 hrs followed by simultaneous addition of 4sU +/- 10 μM colchicine for collection at 2-, 4- and 6-hours post-treatment alongside a -4sU control. All fold changes were calculated relative to the -4sU untreated control for each siRNA KD condition (following normalization to GAPDH control). mRNA half-lives in untreated (black) versus colchicine-treated cells (red) were calculated using a single-phase exponential decay model (confidence intervals provided in Table 1 beneath Roadblock-qPCR methods); n = 3 biological replicates. Error bars indicate mean+/-SD. (G) Graphical summary of CCR4-NOT complex regulation of tubulin transcript levels constitutively, and upon increase in soluble tubulin levels. Graphics generated using BioRender.

## Description

Microtubules, cytoskeletal components underlying intracellular transport, motility and morphology, are non-covalent polymers that self-assemble from αβ-tubulin heterodimers. Microtubule growth rate and abundance are directly dependent on the concentration of these heterodimers (Mitchison & Kirschner, 1984). Cells possess a widely conserved feedback mechanism to monitor and maintain tubulin levels termed tubulin autoregulation. When cells sense an influx of tubulin subunits, such as upon drug-mediated microtubule depolymerization, deflagellation or changes in shape (Baker, Schloss, & Rosenbaum, 1984; Ben-Ze'ev, Farmer, & Penman, 1979; Cleveland, Lopata, Sherline, & Kirschner, 1981; Mooney, Hansen, Langer, Vacanti, and Ingber, 1994; Pachter, Yen, & Cleveland, 1987), they induce co-translational tubulin mRNA degradation. The process requires the first four amino acids of β-tubulin, MREI, which are common to all vertebrate β-tubulin isoforms (David A. Gay, Yen, Lau, & Cleveland, 1987; Pachter et al., 1987; Yen, Gay, Pachter, & Cleveland, 1988), allowing their tandem regulation. Increased soluble tubulin also coordinately triggers the degradation of α-tubulin transcripts, which begin with amino acids MREC (Bachurski, Theodorakis, Coulson, & Cleveland, 1994). While this unique feedback mechanism was first characterized over four decades ago, the molecular players mediating this process remained unknown. Recent identification of the tetratricopeptide repeat domain 5 (TTC5) protein marked the first molecular inroad into the pathway (Lin et al., 2020). Upon microtubule depolymerization, TTC5 binds both the ribosome and the MREI motif as it emerges from the ribosome exit channel (Fig 1A). While this finding resolved how tubulin-translating ribosomes could be specifically recognized, it highlighted the requirement for additional downstream decay effectors.

Tubulin autoregulation necessitates the involvement of cellular RNA decay factors to carry out the final step of tubulin transcript destruction (Fig 1A). Transcript turnover in cells is complex and involves interconnected and regulated processes that act on various features within the mRNA. mRNA turnover may proceed due to activities of specialized enzymes at the 5’ or 3’ end of the transcript *via* deadenylation or decapping, respectively. Decay intermediates can be further processed from 5’ to 3’ by Xrn1 or from 3’ to 5’ by the exosome (Houseley & Tollervey, 2009). In addition to determining bulk mRNA decay, certain pathways or individual factors within these pathways can be targeted to specific transcripts by *cis* (sequence or structure-specific features) or *trans* (through interaction with adaptor proteins) regulatory factors, or through activation of mRNA surveillance pathways. To assess which, if any, of these decay pathways is responsible for the targeted decay of tubulin transcripts during the tubulin autoregulatory response, we selected a panel of genes representing discrete categories of RNA turnover: key genes associated with 3’ to 5’ decay through deadenylation (poly(A) specific ribonuclease subunit PAN2 [PAN2], poly(A)-specific ribonuclease [PARN], CCR4-NOT transcription complex subunit 1 [CNOT1]) as well as the exosome complex (DIS3 exosome endoribonuclease and 3’-5’ exoribonuclease [DIS3], DIS3 like exosome 3’-5’ exoribonuclease [DIS3L]) (Passmore & Coller, 2022; Tomecki et al., 2010) and 5’ to 3’ decay factors such as decapping mRNA 2 [DCP2] and 5’-3’ exoribonuclease 1 [XRN1] (Li, Song, & Kiledjian, 2011; Liu & Moss, 2016). We also prioritized factors related to ribosome stalling and collision-sensing (NEDD4 Binding Protein 2 [N4BP2], General Control Non-depressible 1 [GCN1], Endothelial Differentiation Related Factor 1 [EDF1], Zinc Finger Protein 598 [ZNF598]) (D'Orazio et al., 2019; Sinha et al., 2020; Veltri, D’Orazio, & Green, 2020) as early studies demonstrated the reliance of tubulin autoregulation on active translation and reported enhanced tubulin transcript degradation at low levels of cycloheximide and emetine (D. A. Gay, Sisodia, & Cleveland, 1989) known now to trigger ribosome collisions (Juszkiewicz et al., 2018). We knocked down each gene by siRNA transfection (for validation *via* RT-qPCR see Extended Figure) or utilized previously characterized knock-out lines and measured β-tubulin (TUBB) transcript levels upon colchicine-induced microtubule depolymerization ( Fig. 1B ).

While knock-down of most factors has no significant effect on colchicine-induced tubulin autoregulation (which reduces TUBB transcripts to ~35% of the level in untreated cells), CNOT1 knock-down results in complete loss of tubulin decay, comparable to that upon knock-down of known pathway effector TTC5 (Fig 1B). CNOT1 is the central scaffold of the CCR4-NOT (Carbon Catabolite Repression 4-Negative On TATA-less) complex which is responsible for both global deadenylation functions as well as targeted degradation of transcripts through specific adaptor proteins (Hagkarim & Grand, 2020; Passmore & Coller, 2022; Shah et al., 2024; Shirai, Suzuki, Morita, Takahashi, & Yamamoto, 2014). The complex is organized into three main functional modules: an N-terminal domain comprised of subcomplex CNOT10 and 11, a central catalytic domain comprised of CCR4 (CNOT6 or 6L) and Caf1 (CNOT7 or 8) which possess deadenylase activity with differing specificities (Raisch et al., 2019; Yi et al., 2018) and a C-terminal or “Not” module composed of CNOT2, CNOT3 and the transiently associated CNOT4 subunit (an E3 ubiquitin ligase), which function together in the detection of stalled or slowed ribosomes (Absmeier et al., 2022; Allen et al., 2021; Buschauer et al., 2020; Zhu, Cruz, Zhang, Erzberger, & Mendell, 2024) (Fig 1C). Direct interactions between CNOT1 and DDX6, and CNOT9 and TNRC6 can alternatively link CCR4-NOT complex activity to miRNA-dependent repression (Chen et al., 2014; Mathys et al., 2014). To gain further insight into which subfunctions of the complex are required for its role in tubulin autoregulation, we performed siRNA knock-down of each component in the complex (with combinatorial knock-down of potentially redundant catalytic components CNOT6/6L and CNOT7/8) and assayed for tubulin autoregulation upon colchicine-induced microtubule depolymerization. While we observed partial loss of tubulin autoregulation upon knock-down of several CCR4-NOT components, complete loss of tubulin decay upon CNOT1 knock-down was recapitulated only upon reduction of the factors comprising the N-terminal module, CNOT10 and CNOT11 (Fig 1D). These subunits have recently emerged as a key interaction platform for the targeted recruitment of cellular factors (Mauxion et al., 2022; Mauxion, Prève, & Séraphin, 2013) supporting a model in which this subcomplex serves as an adaptor module for direct or indirect recruitment to TTC5-associated tubulin-translating ribosomes (Fig 1G). We also note that knock-down of most CNOT1 complex components (with exception of CNOT6/6L and CNOT9) results in a baseline increase in tubulin transcript levels relative to the non-targeting control, even in the absence of microtubule depolymerization and even for subunits that had no impact on the autoregulation readout (Fig 1E). This is consistent with the canonical contribution of the CCR4-NOT complex to bulk mRNA turnover (Gillen et al., 2021; Passmore & Coller, 2022) and suggests that the CCR4-NOT complex in addition to its targeted role in tubulin autoregulation also acts constitutively in tubulin transcript turnover (Fig 1G).

While best known for its role in mRNA decay, the CCR4-NOT complex has also been shown to mediate transcriptional repression. We reasoned it could therefore have both direct and/or indirect effects on tubulin transcript levels (Collart & Struhl, 1994; Kruk, Dutta, Fu, Gilmour, & Reese, 2011; Winkler, Mulder, Bardwell, Kalkhoven, & Timmers, 2006). To distinguish between these possibilities and obtain finer resolution into the kinetics of tubulin decay in both untreated and colchicine-treated cells, we utilized Roadblock-qPCR (Watson, Park, & Thoreen, 2021; Watson & Thoreen, 2022). This method uses uridine-analog (4SU) incorporation and adduct addition to distinguish between newly transcribed and existing RNA pools for subsequent mRNA half-life determination while avoiding the general toxicity associated with the more traditional actinomycin D (“transcriptional shutoff”) approach for determining transcript turnover kinetics. As expected, microtubule depolymerization in control cells (NT) resulted in dramatic enhancement of tubulin transcript decay kinetics with TUBB and TUBA1A mRNA half-lives decreasing from ~ 6.1 to ~ 1.9 hrs and ~ 11.5 to ~ 4.4 hrs, respectively (Fig 1F) indicative of a robust tubulin autoregulation response. The untreated half-lives are consistent with previously-reported values (Bachurski et al., 1994). In contrast, in TTC5 KD cells, this autoregulation-dependent reduction in TUBB and TUBA1A half-lives was lost. Knock-down of CNOT1, 10 and 11 all similarly abolished tubulin transcript decay in response to colchicine treatment, however their knock-down also extended the half-lives of tubulin even in untreated cells. This baseline increase in tubulin transcript abundance was consistent with the increase in steady-state tubulin levels we observed upon knock-down of each of these factors using conventional qPCR ( Fig. 1E ). Our results also align with a recent imaging-based screen in HeLa cells in which CRISPR knock-out of CNOT1 and more specifically CNOT10 and 11 strongly increased cellular tubulin intensity even in the absence of microtubule depolymerization (Funk et al., 2022).

In addition to characterizing the decay kinetics of TUBB and TUBA1A, we also assessed the turnover of myc mRNA, a transcript serving as a positive control for rapid turnover (Jones & Cole, 1987). Interestingly, myc half-life increases dramatically upon knock-down of CNOT1 but not upon knock-down of CNOT10 or CNOT11, suggesting it is a target of the CCR4-NOT complex but independent of CNOT10 and 11 (Fig 1F). This observation highlights the modular nature of the CCR4-NOT complex (Ogami, Hosoda, Funakoshi, & Hoshino, 2014) and a specialized role for CNOT10 and 11 in targeting specific transcripts for degradation.

During preparation of this work, the Hegde group similarly identified the CCR4-NOT complex and factors CNOT10 and 11 as the mediators of tubulin autoregulation through an iterative proximity-labeling approach (Höpfler et al., 2023). The protein SCAPER was also uncovered as the molecular bridge between tubulin-translating ribosome recognition and decay by binding to both TTC5 and CNOT11. Our work is a valuable corroboration of these findings using an entirely independent and complementary approach of RNA-decay pathway siRNA knock-down screening while also providing evidence that other canonical decay pathways are dispensable for this process. Together, these studies demonstrate how a general RNA processing complex can be specialized to specific substrates in a context-dependent manner and identify additional molecular handles for exploring the role of tubulin and microtubule homeostasis in cells and tissues.

## Methods

**Mammalian cell line culture and generation:** All HEK-293 cells were cultured in DMEM+Glutamax (ThermoFisher 10564029) supplemented with 10% FBS (Life Technologies A5209402) at 37 °C and 5% CO2. DCP2-/- KO and WT HEK-293 derivative lines were generated and characterized as previously described and obtained courtesy of Mergerditch Kiledjian (Mauer et al., 2017). XRN1-/- KO and WT A549 lines were generated and characterized as previously described (Liu & Moss, 2016) obtained courtesy of Bernard Moss and maintained in RPMI media (ThermoFisher 11875093) supplemented with 10% FBS.

**Drug treatments:** Microtubule depolymerization was performed by administration of colchicine (Sigma C9754) resuspended in water as a 0.1 mM stock and diluted to a final concentration of 10 μM.

**siRNA knock-down in HEK-293:** For siRNA knock-downs, 3.75x10 ^4^ HEK-293 cells were reverse-transfected with Silencer Select siRNAs (reconstituted in water and stored at 20 μM stock solution) using Lipofectamine RNAiMAX (ThermoFisher 13778150) to a final concentration of 40 nM according to manufacturer instructions. Where double-knockdowns (ex. CNOT6/6L, and CNOT7/8) or multiple siRNAs targeting the same gene (ex. PAN2, PARN) were employed, both siRNAs were included at a final concentration of 40 nM. For tubulin autoregulation experiments, at approximately 72 hours post-transfection, cells were either left untreated or treated with colchicine (10 μM) by spiking in an additional 100 ul media and collected at four hours post-treatment for subsequent RNA extraction and quantification. Four hour colchicine treatment was selected based on a timecourse of tubulin autoregulation in HEK-293s and to minimize toxicity in addition to siRNA gene-specific knock-down effects. Silencer select siRNAs (ThermoFisher) used in this study include Negative Control No. 1 (4390843), TTC5 (s40806), CNOT1 (s22842), CNOT10 (s24720), CNOT11 (s30995), CNOT2 (s226689), CNOT3 (s9628), CNOT4 (s9631), CNOT6 (s33101), CNOT6L (s48341), CNOT7 (s26637), CNOT8 (s225112), CNOT9 (s17424), N4BP2 (s31353), DDX6 (s4010), GCN1 (s21626), ZNF598 (s56951), EDF1 (s16609), PAN2 (s19252 and s19253), PARN (s10048 and s10047), DIS3 (s22607), DIS3L (s41866).

**RNA extraction, cDNA preparation and RT-qPCR:** All RNA extractions were carried out using the Zymo Direct-zol RNA Mini- (R2053) or MicroPrep (R2063) Kits according to manufacturer’s instructions and quantified by nanodrop for generation of cDNA from 500-1000 ng of RNA input using the High-Capacity cDNA Reverse Transcription Kit (Life Technologies 4368814). RT-qPCR was performed using Powerup Sybrgreen MasterMix (ThermoFisher A25777) with the primer pairs listed below (either previously described (Lin et al., 2020) or designed using NCBI PrimerBlast) and run on a QuantStudio 6 Flex Real-time PCR System according to manufacturer’s instructions. Each sample was assayed in technical duplicate. For analysis, cDNA input was normalized (dCt) to GAPDH, and expression fold changes were calculated using the ddCt method to a normalizing control (either the untreated or non-targeting condition).

**Roadblock-qPCR for transcript kinetics measurements:** To assay mRNA transcript turnover kinetics, Roadblock-qPCR was performed as previously described (Watson et al., 2021; Watson & Thoreen, 2022). Specifically, 300,000 HEK-293 cells were reverse transfected with indicated Silencer Select siRNAs (as described above). At 72 hours post-transfection, cells were treated with 400 μM 4-thiouridine (4sU) (Cayman Chemical 16373) with or without commensurate addition of colchicine (10 μM) and cells were harvested at intervals of 2 hrs after addition over a time course of 6 hrs. Cells incubated for the duration of the time course +/- colchicine but without the addition of 4sU were harvested as the normalizing control. RNA was extracted using the Zymo miniprep kit as above, treated with N-ethylmaleimide (NEM) (Sigma E3876) and reisolated using RNAClean XP beads (Beckman Coulter) according to manufacturer instructions. Throughout, steps involving 4SU prior to reaction with NEM were performed in the dark to minimize light exposure. The purified RNA was then converted to cDNA using an oligod(T)18 primer (NEB S1316) and Protoscript II reverse transcriptase (NEB M0368) and quantified by RT-qPCR as above, with 5’-biased gene-specific primers including those for GAPDH and myc as previously described (Watson et al., 2021) and noted in primer table using Powerup Sybrgreen MasterMix according to manufacturer’s instructions. All fold changes were calculated using the ddCt method relative to the -4SU untreated control for each siRNA KD condition (following normalization to GAPDH control). mRNA half-lives were calculated using a single-phase exponential decay model in Graphpad Prism (Table 1).

&nbsp;

&nbsp;

&nbsp;

&nbsp;

&nbsp;

&nbsp;

&nbsp;

&nbsp;

&nbsp;

&nbsp;

&nbsp;

&nbsp;

&nbsp;

**Table:** 

		siRNA KD:				
		NT	TTC5	CNOT1	CNOT10	CNOT11
TUBB mRNA	untrt	6.1 [4.1-10.1] 1.9 [1.5-2.4]	6.3 [4.0-11.6] 4.7 [3.2-7.3]	N/A [N/A] N/A [N/A]	12.4 [9.2-18.2] 29.8 [7.3-N/A]	8.7 [6.9-11.3] 13.5 [8.1-30.7]
	+colch					
TUBA1A mRNA	untrt	11.5 [6.9-26.4] 4.4 [3.3-5.9]	10.7 [7.7-16.4] 9.5 [6.6-15.3]	N/A [N/A] N/A [N/A]	15.9 [9.6-37.1] 24.8[14.5-69.2]	16.7 [11.7-28.9] 15.1 [10.9-23.5]
	+colch					
myc mRNA	untrt	0.4[N/A-0.6] 0.5 [N/A-0.7]	0.4 [N/A-0.7] 0.5 [N/A-0.8]	6.0 [3.6-11.4] 8.9 [5.1-21.2]	0.5 [0.4-0.6] 0.5 [0.4-0.7]	0.5 [0.4-0.6] 0.7 [0.5-0.8]
	+colch					

Table 1. Half-lives with confidence intervals [CI] for TUBB, TUBA1A and myc mRNA upon indicated siRNA knock-down.

**Statistical Analysis:** Statistical analysis was performed using GraphPad Prism 9. Single, double, triple and quadruple asterisks indicate p < 0.05, p < 0.01, p < 0.001 and p < 0.0001 respectively, ns=non-significant for p > 0.05.

## Reagents

RT-qPCR Primer Pairs :

**Table:** 

Gene	Forward (5'->3')	Reverse (5'->3')	
TTC5	GGCTTCACCGAATTCAGCAC	TGAGGCTTCCCATTCACCAC	
GAPDH	AACATCATCCCTGCCTCTACTGG	GTTTTTCTAGACGGCAGGTCAGG	
DIS3	TTGCATCATACAGCGAGTGG	GCCAAGTCTTTGGCTGAGTC	
DIS3L	AGAAGGTGCTGCTGCTGAG	ACTTTCCAGTCTGGGATCACG	
N4BP2	CCGCTCCAGAAGCAGTAAGAA	ATCTTGCACAATCCAGCAGT	
GCN1	AGCAAAGTCAAGCCTCCGAA	CCAGCCAGTCCTTTCACGAT	
EDF1	AAGGTGATCCAGCAAGGTCG	CCTTCTCGATGGGCTTTCCA	
ZNF598	TGCTCTACCAAGATGCGGG	CCTTGCGCGAGTACCACTT	
PARN	CAAGCTCAGTGTCGGAAATC	TTTTACAGCTCCCAGCACAG	
PAN2	TCTTCCCGTCCAGGAATCAG	AGGATGAGTAGCGCTCCAAG	
DDX6	TAAAACAGCATGAGCACGGC	GTGGAGGTCACATCCGAAGTT	
CNOT1	CCAGTAGGTGGTCTTGGCAC	CTGAGACAAGTCCGAAGGTTTC	
CNOT2	TAGCCCCAGGGAAACGGTAG	GTCGTGTCACAGGAAGGACC	
CNOT3	CCGGGGCGCGAGAAAAAG	TGAGGCAGCGATCAATCTCA	
CNOT4	ACAGCAAACCCCACCTCAAA	GCCAGTGTGGAGGATTGTCA	
CNOT6	ACAGAACAACCACCTCCAAGG	TGTAGTCCCAGTTTAGCGCC	
CNOT6L	TAGGACCGAGAGTGTTGGGAA	ATGTACTTAGGCTCCGCACTC	
CNOT7	CTGGGCGAGAGGTGTCTATG	AGATGCCAAGCATCAAAATGTTA	
CNOT8	ATCCGGGGTAGAGGGAAAAGA	GTTTTACAGTGCAAGCCAGACA	
CNOT9	GTCCGGCTGTGGAAGAGAG	GACTGGTGTGCTGTCAAGGT	
CNOT10	CGTCTGCCCACTCCTCTAGC	ACTGACCTGTGCCTTCATGTT	
CNOT11	TTCAAAAAGACGCCTCGCCA	GAGGCGGTGGACGAATAAAC	
TUBB	GAAGCCACAGGTGGCAAATA	CGTACCACATCCAGGACAGA	
TUBA1A	CCACAGTCA TTGA TGAAGTTCG	GCTGTGGAAAACCAAGAAGC	
MYC	CGTCCTCGGATTCTCTGCTC	GCTGCGTAGTTGTGCTGATG	
GAPDH (5')	TTCTTTTGCGTCGCCAGCCGA	ACCAGGCGCCCAATACGACCA	

## Data Availability

Description: Validation of siRNA knock-down efficiency for RNA decay and CCR4-NOT complex factors. RT-qPCR for each gene-of-interest to assess degree of knock-down relative to a NT control siRNA at time of colchicine treatment, 72 hrs post-transfection. Data are graphed as the fold change relative to the NT control condition for each siRNA KD (preceded by normalization to housekeeping gene GAPDH). . Resource Type: Image. DOI: https://doi.org/10.22002/2j04v-t2c81
